# Costs of Community-Based Viral Hepatitis Screening in Cameroon Using Point-of-Care Technologies

**DOI:** 10.1093/ofid/ofae378

**Published:** 2024-07-03

**Authors:** Nkengeh N Tazinkeng, Amir M Mohareb, Akwi W Asombang, Emily P Hyle

**Affiliations:** Department of Population Health Research, Health Education and Research Organization, Buea, Cameroon; Division of Gastroenterology, Massachusetts General Hospital, Boston, Massachusetts, USA; Department of Research, Pan-African Organization for Health, Education and Research, Manchester, Missouri, USA; Division of Infectious Diseases, Massachusetts General Hospital, Boston, Massachusetts, USA; Medical Practice Evaluation Center, Massachusetts General Hospital, Boston, Massachusetts, USA; Department of Medicine, Harvard Medical School, Boston, Massachusetts, USA; Division of Gastroenterology, Massachusetts General Hospital, Boston, Massachusetts, USA; Department of Research, Pan-African Organization for Health, Education and Research, Manchester, Missouri, USA; Department of Medicine, Harvard Medical School, Boston, Massachusetts, USA; Division of Infectious Diseases, Massachusetts General Hospital, Boston, Massachusetts, USA; Medical Practice Evaluation Center, Massachusetts General Hospital, Boston, Massachusetts, USA; Department of Medicine, Harvard Medical School, Boston, Massachusetts, USA

**Keywords:** Cameroon, costs, hepatitis B, hepatitis C, point-of-care

## Abstract

This cost analysis of a community-based viral hepatitis screening program in Cameroon found an investment of $3.52 per person screened, $50.63 per new diagnosis of hepatitis B, $159.45 per new diagnosis of hepatitis C, and $47.97 per new diagnosis of either hepatitis B or C.

Approximately 350 million people are living with hepatitis B virus (HBV) and hepatitis C virus (HCV), which results in 1.3 million deaths each year [1]. Treatment for viral hepatitis can be cost-effective as it prevents downstream hepatic complications, such as cirrhosis and hepatocellular carcinoma [2, 3]. However, most people with viral hepatitis are unaware of their diagnosis [1]. Given the benefits of early diagnosis and treatment, community-based screening of HBV and HCV may be preferred as a public health strategy over symptom-driven testing, which delays diagnosis until later in the course of disease.

Cameroon is an HBV-endemic country in sub-Saharan Africa that is currently scaling up a viral hepatitis elimination program. The overall HBV prevalence estimates range from 6.8% to 11.2% [4, 5]. Fewer than 2% of the estimated 1.6 million people living with HBV are diagnosed [5]. HBV prevalence varies between rural (up to 13.3%) and urban (up to 9.0%) areas and by year of birth, given the introduction of universal childhood HBV immunization in 2005 [4]. HCV prevalence in Cameroon also varies by year of birth, with a high prevalence (>50%) in people who were born before 1945 [6].

Point-of-care testing is a feasible, economically efficient, and acceptable means of facilitating community-based screening for infections [7, 8], including chronic viral hepatitis [9, 10]. However, few studies report the costs of implementing viral hepatitis screening programs in sub-Saharan Africa. Cost estimates are essential for public health planning and to assist decision-makers as they weigh the trade-offs of investing limited healthcare resources in viral hepatitis elimination programs. Our objective was to estimate the costs and resource utilization required for a community-based viral hepatitis screening program in Cameroon.

## METHODS

We implemented a 9-day community-based viral hepatitis screening program from 28 February 2021 to 15 May 2021 in 4 health areas (ie, subdistricts) in the South West Region of Cameroon [11]. This previously described hepatitis screening program detected 65 new HBV diagnoses, 22 new HCV diagnoses, and 3 HBV/HCV coinfections among 1144 people screened [11]. We undertook a costing analysis of this screening program by collecting and analyzing all costs associated with its design and implementation.

We used a costing framework to estimate resource utilization across the following categories: personnel, transportation, consumable items, social mobilization, and capital items [12]. We estimated personnel costs using the market price method [13]. We valued time spent by people performing community screening (“screeners”) at the estimated salary of community health workers (US$3.00/hour). Eleven screeners underwent 4 hours of training with the project lead ($6.50/hour) and laboratory staff ($5.50/hour), who conducted the technical training. The project lead and screeners together implemented the screening program at community sites using the public bus system for transportation. We report transportation costs stratified by the subdistrict and the day of the screening program. Consumable items were divided into field items and laboratory items. Field items included office supplies, gauze, lancets, alcohol swabs, biohazard waste receptacles, and personal protective equipment for the testers. Laboratory items included test strips and buffer reagents. Capital costs included the time spent planning the program by the project lead. We categorized the resources into fixed and variable costs; fixed costs did not vary with the scale of the program, whereas variable costs increased with each additional participant. We converted costs from 2021 Central African francs to 2023 US dollars ($).

We estimated the costs per participant in the screening campaign by dividing total costs by the total number of participants screened. We determined the costs per new HBV and/or HCV diagnosis by dividing the costs by the number of positive test results during the campaign. We determined the marginal costs of the screening program, defined as the costs of screening each additional participant, by dividing the variable costs of the program by the number of people screened.

Written patient consent was obtained for the parent study, which received ethical approval from the Faculty of Health Sciences Institutional Review Board (protocol number 1281-02) of the University of Buea.

## RESULTS

The total cost of the screening program was $4029.29, comprising personnel ($2173.00 [54.0%]), transportation ($93.58 [2.3%]), consumable items ($1549.36 [38.5%]), social mobilization ($63.35 [1.6%]), and capital costs ($130.00 [3.7%]) (Table 1). The fixed costs of the program included training time with laboratory staff and project lead ($70.00), program implementation by the project lead ($351.00), and social mobilization ($63.00). The variable costs of the program included training time for the screeners ($132.00), implementation time for the screeners and project lead ($1620.00), transportation costs ($93.58), and consumable items ($1549.36).

**Table 1.** Costs of a Viral Hepatitis Screening Program by Category of Expenditure in Buea, Cameroon, 2021

Category	Unit Cost	Cost (2023 US Dollars)	Fixed/Variable
Personnel	($/hour)		
Training (No. of person-hours)			
Project lead (4)	$6.50	$26.00	Fixed
Screeners (44)	$3.00	$132.00	Variable
Laboratory staff (8)	$5.50	$44.00	Fixed
Implementation (No. of person-hours)			
Registration (108)	$3.00	$324.00	Variable
Specimen testing (216)	$3.00	$648.00	Variable
Result and counseling (108)	$3.00	$324.00	Variable
Data entry (108)	$3.00	$324.00	Variable
Team lead (54)	$6.50	$351.00	Fixed
Subtotal—personnel	…	$2173.00	…
Transportation for staff and supplies			
Muea			
Day 1	…	$11.77	Variable
Day 2	…	$9.05	Variable
Day 3	…	$5.43	Variable
Day 4	…	$18.10	Variable
Molyko			
Day 4	…	$9.05	Variable
Day 5	…	$9.05	Variable
Bokwango			
Day 6	…	$7.24	Variable
Buea			
Day 7	…	$10.14	Variable
Day 8	…	$7.24	Variable
Day 9	…	$6.52	Variable
Subtotal—transportation	…	$93.58	…
Consumable	($/box)		
Field items			
Lancet (12 boxes)	$12.67	$152.04	Variable
Gauze (2 boxes)	$9.05	$18.10	Variable
Alcohol swab/cleaning (6 boxes)	$1.81	$10.86	Variable
PPE (12 boxes)	$9.05	$108.60	Variable
Laboratory items			
HBV strips/reagents (24 boxes)	$21.72	$521.28	Variable
HCV strips/reagents (24 boxes)	$30.77	$738.48	Variable
Subtotal—consumables	…	$1549.36	…
Social mobilization costs			
Radio announcements (2 announcements)	$27.15	$54.30	Fixed
Banners and signs (1 banner)	$9.05	$9.05	Fixed
Subtotal—social mobilization	…	$63.35	…
Capital/overhead costs			
Preparation and planning (20 h)	$6.50	$130.00	Fixed
Subtotal—capital	…	$130.00	…
Total costs	…	$4029.29	…

Abbreviations: HBV, hepatitis B virus; HCV, hepatitis C virus; PPE, personal protective equipment; US, United States.

The program cost $3.52 per participant screened, $50.63 per new diagnosis of HBV, $159.45 per new diagnosis of HCV, and $47.97 per new diagnosis of either HBV or HCV. The marginal cost of the program was $2.97 per each additional participant.

## DISCUSSION

In this analysis of a community-based viral hepatitis screening program in Buea, Cameroon, we found that the overall program cost $4029.29 to screen 1144 people, amounting to $3.52 per person screened. Personnel and consumable items were the most costly categories of resource utilization. These results demonstrate expenditures necessary to implement a community-based viral hepatitis screening program as well as the yield of this investment in diagnosing new HBV and HCV in asymptomatic participants. These findings are important for health systems in sub-Saharan Africa as they scale up viral hepatitis screening and treatment programs, which can be cost-effective as they prevent early morbidity and mortality related to hepatic complications [2].

Limited data are available regarding the costs of community-based viral hepatitis screening in endemic countries. Compared to a recent study of HBV screening in Vietnam, our serosurvey included fewer people (1144 vs 2072 participants) yet had a 10-fold lower cost per participant screened ($3.52 vs $38.70 per participant) [12]. This large difference is the result of variations in staff salaries and consumable costs between these 2 different HBV-endemic countries. That study also tested more children—46.2% of their participants were under the age of 15—which may have increased the personnel time and resources required per participant in their study, whereas all participants in our study were >15 years old.

Even though the costs of this screening program are lower than previously reported, population-based viral hepatitis screening programs are generally underresourced in low- and middle-income countries [1]. Implementation of such programs may be more likely to be prioritized by funders by building on existing opportunities (eg, incorporating viral hepatitis screening within existing screening programs for human immunodeficiency virus and other sexually transmitted infections). Implementation of point-of-care hepatitis D testing may also improve the efficiency of the program while enhancing the care of people living with HBV in endemic countries such as Cameroon [14].

Viral hepatitis screening must be accompanied by investments in linkage to care and access to treatment [1]. For HCV, early diagnosis yields substantial health benefits if linkage to curative treatment with direct-acting antivirals is achieved. However, for HBV, the additional cost of determining treatment eligibility is a further barrier to treatment initiation in many parts of sub-Saharan Africa, including Cameroon. Though updated World Health Organization (WHO) guidance on HBV treatment aims to simplify treatment eligibility [15], determining HBV treatment eligibility still requires several diagnostic tests (ie, assessment of aminotransferase elevation, HBV DNA level, and liver injury with liver elastography, ultrasound, or biopsy). The Ministry of Health in Cameroon has recently established viral hepatitis treatment centers in all 10 regions of the country, which includes subsidization of medication costs: Out-of-pocket expenses for patients with HBV are now $3.10 per month for tenofovir, and a full treatment course for HCV is free of charge to patients [16]. However, linkage to care still depends on patients’ capacity to travel to and access treatment centers and, in the case of HBV, to pay for unsubsidized laboratory tests. Improving point-of-care testing capacity for HBV DNA and simplifying HBV treatment initiation criteria may reduce costs of determining treatment eligibility for people known to be hepatitis B surface antigen positive [10].

The availability of qualified personnel varies across different countries and regions, and the costs of training and staff salaries depend on the regional context, which limits the generalizability of our findings. Local factors related to transportation, public awareness, and acceptability of screening further limit generalizability. Screening program yield depends on the capacity of personnel to enroll and test additional participants for each day of testing; expanding this program could entail additional training and implementation costs. We did not include reference testing for HBV and HCV but used a point-of-care assay with a high reported sensitivity and specificity in community testing [11].

In conclusion, we found that this community screening program for HBV and HCV among 1144 persons in Cameroon cost $4029.29, or $3.52 per person screened and $47.97 per person newly diagnosed with HBV or HCV. These cost estimates, which are lower than published findings from other endemic settings, can help public health authorities as they implement screening programs to meet the WHO viral hepatitis elimination targets. Specifically, these findings can be incorporated into cost-effectiveness analyses, which may motivate policymakers to invest in viral hepatitis screening programs to prevent the downstream morbidity, mortality, and costs of chronic hepatitis B and C.

## Notes

***Data availability.*** Data from this study are available from the corresponding author upon reasonable request.

***Financial support.*** A. M. M. is supported by the National Institutes of Health (grant number K01AI166126).
